# Factors associated with death in COVID-19 patients over 60 years of age at Kinshasa University Hospital, Democratic Republic of Congo (DRC)

**DOI:** 10.11604/pamj.2022.41.330.32602

**Published:** 2022-04-22

**Authors:** Ben Bepouka, Madone Mandina, Murielle Longokolo, Nadine Mayasi, Ossam Odio, Donat Mangala, Yves Mafuta, Jean Robert Makulo, Marcel Mbula, Jean Marie Kayembe, Hippolyte Situakibanza

**Affiliations:** 1 Infectious and Tropical Diseases Unit, Kinshasa University Hospital, Kinshasa, Democratic Republic of the Congo,; 2 Nephrology Unit, Kinshasa University Hospital, Kinshasa, Democratic Republic of the Congo,; 3 Pneumology Unit, Kinshasa University Hospital, Kinshasa, Democratic Republic of the Congo

**Keywords:** COVID-19, advanced age, mortality, risk factors, Kinshasa, Democratic Republic of Congo

## Abstract

**Introduction:**

the objectives of the present study were to determine the mortality rate in patients over 60 years of age with COVID-19 and to identify risk factors.

**Methods:**

the present historical cohort study took place at the Kinshasa University Hospital (KUH), DRC. Older patients admitted from March 2020 to May 2021 and diagnosed COVID-19 positive at the laboratory were selected. The relationship between clinical and biological risk factors, treatment, and in-hospital mortality was modeled using Cox regression.

**Results:**

of two hundred and twenty-two patients at least 60 years old, 97 died, for a mortality rate of 43.69%. The median age was 70 years (64-74) with extremes of 60 to 88 years. Low oxygen saturation of < 90% (aHR 1.69; 95% CI [1.03-2.77]; p=0.038) was an independent predictor of mortality. The risk of death was reduced with corticosteroid use (aHR 0.54; 95% CI [0.40-0.75]; p=0.01) and anticoagulant treatment (aHR 0.53; 95% CI [0.38-0.73]; p=0.01).

**Conclusion:**

mortality was high in seniors during COVID-19 and low oxygen saturation on admission was a risk factor for mortality. Corticosteroid therapy and anticoagulation were protective factors. These should be considered in management to reduce mortality.

## Introduction

Several health facilities in Wuhan, Hubei Province, China, reported patients with pneumonia of unknown origin by the end of December 2019 [1]. The majority of the illnesses among the initial 27 hospitalized patients were related to the Huanan Seafood Wholesale Market, a wet market in downtown Wuhan that sells not just seafood but also live animals, including poultry and wildlife [2,3]. Following that, the disease quickly spread from Wuhan to other regions of China and the rest of the world. Coronavirus type 2 disease with severe acute respiratory syndrome became a new disease (SARS). Finally, SARS-CoV-2 will be the disease's name (i.e. Coronavirus Disease -19, COVID-19, 19 to recall the year 2019). The COVID-19 pandemic has been proclaimed by the World Health Organization (WHO) on March 11, 2020 [4]. COVID-19 has reached 42,385,317 people globally as of July 22, 2021, when this article was written, with 650,684 deaths. In Africa, 4,634,617 infections have resulted in 109,711 fatalities, a mortality rate of 0.23 percent [5].

According to data from China, old age and underlying sickness are two major risk factors for COVID-19-related disease and death [6-8]. Although the majority of reported COVID-19 cases were mild. The mortality rate was just 2.3 percent, and more than 80 percent of deaths occurred in those over 60, with the mortality rate rising rapidly from 3.6 percent in those aged 60-69 to 14.8 percent in those aged 80 [9,10]. In other parts of the world, such as the United States of America (USA) and Europe, COVID-19 has caused a disproportionate hazard to the elderly [11,12]. Several studies in Africa have also found that age is a predictor of mortality [13-17]. Advanced age has been demonstrated to be a negative prognostic factor in DRC investigations [18-20]. Not all elderly individuals with COVID-19, however, die. As a result, identifying death risk factors is critical. Hence, the importance of identifying risk factors for death. The objectives of the present study were to determine the mortality rate of patients over 60 years of age and to identify risk factors for severity and mortality in these patients.

## Methods

The current study was a retrospective cohort. It spanned the months of March 2020 and May 2021. Kinshasa University Hospital, a big regional hospital in Kinshasa, DRC, was the site of this research. This study enrolled all patients hospitalized with confirmed COVID-19 with outcome (death or discharge). The World Health Organization (WHO) interim guideline was used to diagnose COVID-19 patients [21]. Patients who were consulted during the study period and were admitted as confirmed cases of COVID-19 were included in this study. Patients who have been transferred or who have escaped have not been listed.

**Data collection:** every hospitalized patient who meets the inclusion criteria is offered the chance to participate in the survey on a regular basis. The sociodemographic, clinical, biochemical, and co-morbidities of the patients were acquired from their medical records. The data was evaluated by a team of doctors who were well-versed in the subject.

**Confirmation of diagnosis:** confirmed cases were defined by the positive findings in reverse-transcriptase- polymerase chain reaction (RT-PCR) assay of throat swab specimens [22]. Pharyngeal swab specimens were collected from each patient for viral nucleic acid detection of SARS-CoV-2 using RT-PCR assay as previously described [23].

**Statistical analysis:** continuous variables were assumed to be normal using histograms. Categorical variables were presented as frequencies and percentages, normally distributed continuous variables as mean with standard deviation (SD), while non-normal continuous variables were presented as median with interquartile range (IQR). Chi-square and Wilcoxon rank sum tests were used to determine differences in characteristics between surviving and deceased presenting patients. To determine risk factors for death, we used unadjusted odds ratios (OR). Risk factors that emerged in univariate were imputed in Cox regression models to determine those that were predictors of death. Adjusted hazard ratios (aHR) and 95% confidence intervals (CI) were calculated. The value of p < 0.05 was considered the threshold for statistical significance. Data analysis was performed using STATA version 14 (StataCorp, USA).

**Ethical considerations:** the study was approved by the University of Kinshasa School of Public Health's Ethics Committee (ESP/CE/179/2020). The principle of anonymity and confidentiality was maintained in the study. No conflict of interests was declared in the conduct of this study.

## Results

**Figure 1:**
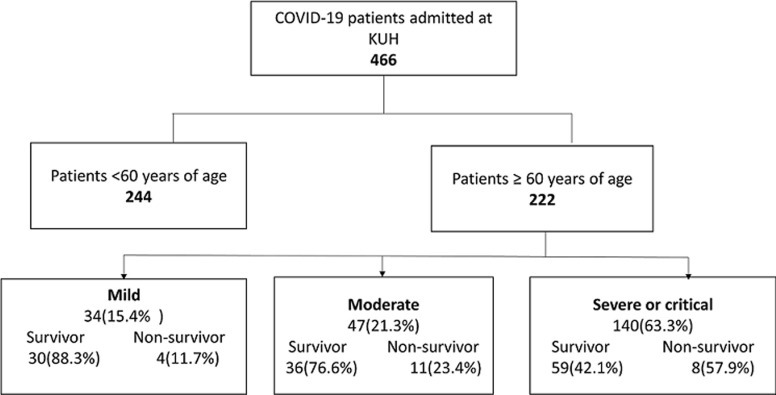
flow chart of COVID-19 patient´s selection

During the study period, 466 patients were hospitalized for COVID-19. These included 222 patients aged 60 years or older and 244 patients under 60 years of age. Of the 222 patients aged 60 years or older hospitalized, 97 died, for a mortality rate of 43.69% (Figure 1). The median age was 70 years (64-74) with extremes of 60 and 88 years. The age group of 60-74 years was predominant (77%). The proportion of men was in the majority (72%). Comorbidities were present in 6.42% of cases. The most common comorbidities were hypertension, followed by diabetes mellitus, cardiovascular disease, obesity and tuberculosis. According to the WHO clinical staging, 63% of the patients were severe (Table 1).

**Table 1:** general characteristics of patients at least 60 years of age hospitalized at the KUH from March 2020 to May 2021

Characteristics	All patients (n= 222)	Survivor (n= 125)	Non survivor (n= 97)	p-value
**Demographic and clinical**				
Age median years (IQR)	70 (64-74)	70(64-74)	69(64-74)	0.979
Age group(years), n (%)				0.979
60-74	170(76.92)	96(76.80)	74(77.08)	
75-79	34(15.38)	19(15.20)	15(15.63)	
>=80	17(7.69)	10(8.00)	7(7.29)	
Gender, n (%)				0.559
Male	159(71.95)	88(70.40)	71(73.96)	
Female	62(28.45)	37(29.60)	25(26.04)	
missing				
**Comorbidities, n(%)**				0.216
Yes	149 (67.42)	80(64.00)	69(71.88)	
No	72(32.58)	45(36.00)	27(28.13)	
**HTN, n (%)**				0.127
Yes	115(52.51)	59(47.97)	56(58.33)	
No	104(47.49)	64(52.03)	40(41.67)	
**DM, n (%)**				0.960
Yes	59(27.49)	32(27.35)	26(27.66)	
**No**	153(72.51)	85(72.65)	68(72.34)	
**CVD**				0.422
Yes	7(3.45)	5(4.35)	2(2.7)	
No	196(96.55)	110(95.65)	86(97.73)	
**Tuberculosis**				0.128
Yes	3(1.40)	3(2.46)	0(0.0)	
No	2.2(98.60)	119(97.54)	93(100)	
**Obesity, n(%)**				0.093
Yes	5(2.35)	1(0.83)	4(4.35)	
No	208(97.65)	120 (99.17)	88 (95.65)	
RR	26 (23-32) 96	25 (22-31) 62	28.5 (24-41)34	**0.023**
**Temperature**	(36.8±0.95) 96	(36.64±0.84)62	(37.08±1.1)34	**0.029**
HR	(9.067±13.16)96	(88.9±13.09)62	(93.91±12.86)34	0.074
**Oxygen saturation,n(%)**				**<0.0001**
<90%	144 (65.45)	63 (50.81)	81 (84.38)	
90-94%	49 (19.55)	34 (27.42)	9 (9.38)	
>=95%	33 (19.55)	27 (21.77)	6(6.25)	
**Severity**				**<0.0001**
Mild	34(15.38)	30 (24)	4 (4.17)	
Moderate	47 (21.27)	36 (28.84)	11(11.46)	
severe	140 (63.35)	59 (47.2.)	81(84.38)	

**IQR**: interquartile range **HTN**: hypertension **DM** : diabetes mellitus **CVD:**cardiovascular diseases**RR**: respiratory rate **HR**: heart rate

Compared with those who survived, we observed that the deceased patients had higher respiratory rates (p = 0.023), higher mean temperatures and lower saturation (<0.0001) and most were in the severe stage. On the basis of laboratory tests, the decedents had elevated white blood cell count, hypernatremia tendency (0.034), hyperglycemia (0.0013), and elevated AST (0.0067) (Table 2). Therapeutically, the proportion of survivors in those with conventional therapy was higher than in those without conventional therapy. Those who had corticosteroids died less than those who did not (Table 3).

**Table 2:** biologic characteristics of older patients admitted to the COVID-19 treatment center of the Kinshasa University Hospital between March 2020 and May 2021

characteristics	All (N=222)	Survivor (n=125)	Non survivor (n=97)	p
Leucocytes (median, IQR) (/mm3), N	10900 (7300-16000)79	9410(6630-13900)49	13050(9780-17800)30	**0.037**
Lymphocytes (median, IQR) (%), N	15.4(9.5-23.8)33	17.6(10.05-25.9)24	9(4-15.8)9	0.089
Neutrophiles (median, IQR) (%l), N	78.2(6985)33	77.65(66.75-83.45)24	84.1(74.9-89)9	0.105
Monocytes (median, IQR) (%l), N	5(2-6.7)25	5.2(2-6.7)19	3.5(2-6.9)6	0.726
Rapport neutrophile/lymphocytes(median, IQR), N	4.74(3-8.76)33	4.30(2.66-8.37)24	9.34(4.74-22.25)4	0.075
Calcium (median, IQR) (mmol/l), N	1.07(1.02-1.12)52	1.08(1.05-1.14)30	1.06(1-1.1)	0.098
Sodium (median, IQR) (mmol/l), N	139(134-143)55	137.1(133.5-140.5)32	142(135-149)23	**0.034**
Hb (median, IQR) (g/dl), N	12.95(10.8-15.1)80	12.4(10.9-15)49	13.3(10.3-15.2)31	0.996
Glycemia (median, IQR) (mg/dl), N	156(117-208)98	133.5(114-172)58	182(138.5-245.5)40	**0.0013**
Creatinine (median, IQR) (mg/dl), N	1.8(1.04-4.55)67	1.35(1.04-3.4)39	3.15(1.08-8.39)28	0.067
Urea (median, IQR) (mg/dl), N	37.02(7.54-78.1)56	37.24(7.31-59.5)33	33.7(7.78-100)23	0.900
CRP (median, IQR) (mg/l), N	84.5(47.1-130)30	74.94(36.2-130)16	98.5(62-130)14	0.300
ALAT(median, IQR) (UI/L), N	35.28(22.1269.52)39	33.38(19.56-62.54)24	46.3(22.99-84.84)15	0.452
ASAT (median, IQR) (UI/L), N	57,51(43-109.39)39	48.49(37.34-70.34)24	93.7(50.99-214.04)	**0.0067**

**IQR**: interquartile range **Hb**: hemoglobin **CR**P: C reactive protein **ALAT**: alanin aminotransferase **ASAT**: aspartate aminotransferase

**Table 3:** biologic characteristics and treatment of older patients admitted to the Covid-19 treatment center of the Kinshasa University Hospital between March 2020 and May 2021

Characteristics	All	survivor	Non survivor	P-value
pH (median, IQR), N	7.39(7.32-7.45)46	7.39(7.33-7.45)22	7.39(7.31-7.45)24	0.467
Po2 (median, IQR) (mmHg), N	54(42-65)45	48(42-74)22	54(41-64)23	0.891
Pco_2_ (mean, SD) (mmHg), N	(40.08±11.00)45	(39.6±12.71)23	(40.58±9.16)22	0.768
Hco_3_(mean, SD) (mmol/l), N	(24.4±6.75)45	(23.88±4.45)22	(25.01±8.47)23	0.578
**Treatment**				
**Conventional treatment, n (%)**				**0.006**
Yes	200(91.32)	118(95.93)	82(85.42)	
No	19(8.68)	5(4.07)	14(14.58)	
**Traitement anticoagulant, n**				**0.032**
Yes	142(70.65)	86(76.79)	56(62.92)	
No	59(29.35)	26(23.21)	33(37.08)	
**Corticoids, n**				**0.022**
Yes	129(65.48)	79(72.48)	50(56.82)	
No	68(34.52)	30(27.52)	38(43.18)	
**Ventilation mecanique, n**				0.104
Yes	6(2.75)	0(0.0)	6(6.45)	
No	212(97.25)	125(100)	87(93.55)	
**Dialysis, n**				0.963
Yes	9(4.05)	5(4.00)	4(4.12)	
No	213(95.95)	120(96.00)	93(95.88)	

After adjustment for other univariate emerging demographic, clinical, and therapeutic factors, low oxygen saturation of <90% (aHR 1.69; 95% CI [1.03-2.77]; p=0.038) was considered an independent factor associated with mortality in elderly COVID-19 patients. The risk of death was reduced with corticosteroid use (aHR 0.54; 95% CI [0.40-075]; p=0.01) and anticoagulant therapy (aHR 0.53; 95% CI [0.38-0.73]; p=0.01) (Table 4).

**Table 4:** cox regression of factors associated with hazard of death at the COVID-19 treatment center of the Kinshasa University Hospital between March 2020 and May 2021

characteristics	Adjusted Hazard ratio (95% CI)	p-value
**Respiratory rate**		0.620
No	1	
Yes	1.00(0.98-1.03)	
**Temperature**		0.218
No	1	
Yes	0.85(0.66-1.09)	
**Oxygen saturation**		
95%	1	
90 % - 94%	1.73(1.10-2.75)	0.118
< 90%	1.69 (1.03-2.77)	**0.038**
**Leucocytes**		0.39
No	1	
Yes	1.00(0.99-1.00)	
**Na**		0.86
No	1	
Yes	0.99(0.96-1.00)	
**Glycemia**		0.88
No	1	
Yes	1.00(0.99-1.00)	
**ASAT**		0.69
No	1	
Yes	1.00(0.99-1.00)	
**Conventional treatment**		
No	1	0.39
Yes	0.79(0.47-1.30)	
**Corticosteroid**		
No	1	
Yes	0.54(0.40-0.73)	**0.01**
**Anticoagulation**		
No	1	
Yes	0.53(0.38-0.73)	**0.01**

## Discussion

The present study was conducted to determine the mortality rate in COVID-19 patients at least 60 years old and to identify risk factors for severity and mortality. It was found that the mortality rate in this age group was 43.69%. This result is slightly higher than the preliminary data estimated in LTCFs in Italy, where mortality was reported to be as high as 37.4% and, again in the same country, a mortality rate of 32% was also reported. The mortality rate in the present work is much lower than that observed in other countries (49-64%) [23-26]. The median age was 70 years (64-74) with extremes of 60 and 88 years. The age range of 60-74 years was predominant (77%). The proportion of men was the majority (72%). The literature agrees that the main risk factors for severe COVID-19 are advanced age, male gender, obesity, smoking, and comorbid chronic diseases such as hypertension, type 2 diabetes mellitus, and others. Of all these factors listed, age is the most important risk factor for severe COVID-19. The influence of age on the severity of COVID-19 has several hypotheses, including one related to immune factors. This hypothesis encompasses age-related impairment of the immune response and protection against SARS-CoV-2 and immunopathology. The immune response includes humoral immunity (i.e., antibody response) and cell-mediated immunity (CMI). While age-related immunosenescence is thought to weaken immune protection, vaccination strengthens it. Inflammation and the cytokine storm can lead to immunopathology. Not all immune responses are protective, as antibody-dependent enhancement (ADE) in humoral immunity may promote SARS-CoV-2 infection, whereas the Th17 response in MCI may contribute to the cytokine storm. Age-related decreases in physiologic reserve in the respiratory and other organ systems may also contribute to vulnerability. Together, they lead to disproportionate severity of COVID-19 and high mortality in the elderly [27].

Although this vulnerability exists, studies to define factors that impact antibody quality and titer, including neutralization of SARS-CoV-2, reveal that advanced age, male gender, and hospitalization for severe COVID-19 all contribute to a greater antiviral antibody response to SARS-CoV-2 [28]. The most common comorbidities were hypertension, followed by diabetes mellitus, cardiovascular disease, obesity, and tuberculosis. Aging and comorbidities are characterized by a higher basal pro-inflammatory state (inflammaging) related to a progressive inability of the immune system to initiate appropriate responses (immunosenescence) [29]. Age-related gut dysbiosis has also been reported to cause an unbalanced immune response and hyperactive inflammatory phenotype in SARS-CoV-2 infection [30]. In addition, other factors, such as increased endothelial damage and age-related changes in coagulation function, higher density, increased affinity, and different distribution of angiotensin-converting enzyme 2 (ACE 2) and transmembrane serine protease 2 receptors, lower levels of vitamin D [31], and mitochondrial dysfunction have also been proposed as conditions that would increase the severity of the disease in older adults [32]. Despite these limitations, the use of appropriate statistical tools identified comorbidity as an independent factor associated with 30-day in-hospital mortality in patients in the COVID-19 study. However, the present study did not find comorbidities as an independent factor associated with death in the older population [33]. A trend toward hypernatremia (p = 0.034), hyperglycemia (p = 0.0013), and elevated AST (p = 0.0067). Hypernatremia would influence severe COVID-19. Also, identification of SARS-CoV-2 ribonucleic acid (RNA) in the urine of an infected patient shows that the virus can enter the tubular fluid where it can bind to its ACE-2 receptors in the proximal tubule. After binding, SARS-CoV-2 initially enters the cells with a membrane receptor that is functionally removed from the outer site of the membrane. After endocytosis of the viral complex, surface ACE 2 is further downregulated, resulting in unopposed angiotensin II accumulation. Angiotensin II can further decrease ACE 2 expression. Angiotensin II facilitates sodium reabsorption by stimulating sodium-hydrogen exchange in the renal proximal convoluted tubule. Increased renal sodium reabsorption is accompanied by increased renal chloride reabsorption and increased potassium excretion, which can lead to hyperchloremia and hypokalemia. These phenomena actually occur much more frequently in patients with hypernatremia than in those without, supporting the possibility of increased angiotensin II activity [34].

Studies indicate mechanisms that are accentuated in patients with type 2 diabetes mellitus (T2DM). Infection with severe acute respiratory syndrome coronavirus 2 (SARS-CoV-2) can lead to increased levels of inflammatory mediators in the blood, including lipopolysaccharide, inflammatory cytokines, and toxic metabolites. Modulation of natural killer cell activity and IFN? production may increase interstitial and/or vascular permeability to proinflammatory products. In addition, SARS-CoV-2 infection results in increased production of reactive oxygen species (ROS). These effects lead to pulmonary fibrosis, acute lung injury and acute respiratory distress syndrome (ARDS). ROS production and viral activation of the renin-angiotensin-aldosterone system (RAAS) (via increased angiotensin II expression) result in insulin resistance, hyperglycemia, and vascular endothelial damage, all of which contribute to cardiovascular events, thromboembolisms, and disseminated intravascular coagulation (DIC). Infection also causes an increase in the coagulation components fibrinogen and D-dimer, resulting in increased blood viscosity and vascular endothelial damage and associated cardiovascular events, thromboembolism, and DIC [35]. The proportion of survivors in those with conventional therapy was greater than in those without conventional therapy. Recently, hydroxychloroquine sulfate (HCQS) has received significant media and political attention for the treatment and prophylaxis of COVID-19, despite the lack of convincing and unequivocal data on its efficacy and safety in these patients. This has unfortunately, but predictably, led to several controversies and confusions within the medical fraternity, the patient community, and the general public. Based on the available studies, many of which have a high risk of bias, relatively small sample sizes, and abbreviated follow-ups, it is unlikely that HCQS has a significant benefit for COVID-19 patients, but it does have the potential to cause harm, particularly when used in combination with azithromycin for some [36] and this combination would be effective against COVID-19 for others [37].

After adjustment, patients were admitted with low oxygen saturation of < 90% (aHR 1.69; 95% CI [1.03-2.77]; p=0.038). Oxygen saturation was considered a related clinical measure and was also easy to obtain. When it is below 90% on admission, despite oxygen supplementation, it is a stronger risk factor for fatal outcome. Indeed, this measure is a more powerful predictor than multiple measures we have obtained, including the more standard demographic and inflammatory measures reported in previous studies [38,39]. In contrast, other studies have found hypoxemia to be an independent factor in mortality in patients of all ages in a study in Nigeria [40]. The pathophysiology of hypoxemia-related death in COVID-19 patients involved an exacerbation of inflammatory cytokines and immune response with an exacerbation of the cytokine storm, leading to lung damage and persistent hypoxemia, and ultimately to poor outcomes [41]. In low- and middle-income countries, a portable pulse oximeter, which is inexpensive and affordable, can help with early screening to identify patients with hypoxemia, allowing early intervention. Corticosteroid therapy was a protective factor for mortality (aHR 0.54; 95 CI [0.40-0.75]; p = 0.01). The RECOVERY trial showed that patients receiving dexamethasone 6 mg had lower mortality than controls [42], whereas the METACOVID trial found no benefit in 28-day mortality and several secondary outcomes of treatment with methylprednisolone 0.5 mg/kg twice daily [43]. A possible explanation for these divergent results is that the dose of corticosteroids in the latter trial was significantly higher than in the former.

It is possible to speculate that the higher doses of corticosteroids may be harmful, which may offset the benefits observed in the RECOVERY trial. In patients with mild SARS-CoV-1, a randomized controlled trial failed to show a beneficial effect of hydrocortisone administration. Of note, higher viremia was observed in the second and third weeks after infection in the hydrocortisone group than in the control group. Similarly, corticosteroids have been reported to be associated with delayed shedding of the SARS-CoV-2 virus, particularly when high doses are administered. Preliminary data from cohort studies has shown a high incidence of bacterial superinfections and pulmonary aspergillosis in mechanically ventilated COVID-19 patients; both of these complications may be associated with corticosteroid use [44]. Anticoagulation therapy was a protective factor for mortality (aHR 0.53; 95 CI [0.38-0.73]; p=0.01). Anticoagulation has been suggested as a mitigating option in patients with COVID-19 due to the higher risk of macrovascular and microvascular thrombosis. In addition, the anti-inflammatory effect of heparins may be beneficial in this highly inflammatory condition. Furthermore, it is proposed that anticoagulation may block or slow the progression of DIC. While anticoagulation is controversial in classical sepsis, COVID-19 sepsis is a distinct entity as reflected by the difference in coagulation parameters. Therefore, anticoagulation seems to have an important role in the treatment of COVID-19. A recent Chinese study by Tang *et al*. [45] described 449 patients with severe COVID-19 infection and reported reduced mortality with anticoagulation in patients with high D-dimer levels and/or high sepsis-induced coagulopathy (SIC) scores.

Another recent study from New York examined the effect of therapeutic-dose anticoagulation on 2,773 unselected hospitalized patients with COVID-19. The study reported a modest improvement in median survival with the use of anticoagulation. However, this benefit appeared to be significantly greater in mechanically ventilated patients, with a 33.6% reduction in mortality. In-hospital mortality in mechanically ventilated patients was 29.1% and 62.7% for patients who received and did not receive anticoagulation, respectively. The median duration of anticoagulation was 3 days, and longer anticoagulation correlated with better survival. However, D-dimer and CIS were not reported in this study and were not used as decision factors to prompt anticoagulation use. In the said study, it is likely that sicker patients were more likely to receive anticoagulation, as evidenced by the higher number of mechanically ventilated patients in the anticoagulation group. Prospective clinical trials are underway to confirm the survival benefit of anticoagulation in patients with COVID-19 [46].

**Limitations**: the present study involved a small population size. This did not allow us to indicate a clear degree of certainty about the results presented. Some parameters, especially biological ones, were not taken into account. Thus, certain biological markers such as D-dimer, ferritin, troponin, and interleukins were not analyzed.

**Strengths**: on the other hand, the main strength of the present study is that the patients were all infected during a limited period. Thus, this is an incident cohort and the analysis of outcome predictors may be more reliable. Also, to the best of our knowledge, this is the first study to examine the sociodemographic and clinical characteristics, mortality, and prognostic factors of a complete cohort of COVID-19 patients at least 60 years old in sub-Saharan Africa.

## Conclusion

Mortality was high in seniors during COVID-19 and low oxygen saturation on admission was a risk factor for mortality. Corticosteroid therapy and anticoagulation were protective factors. These should be considered in management to reduce mortality.

### What is known about this topic

- Older age is associated with poorer outcomes of SARS-CoV-2 infection;
- Living with someone of working age, living in a care home, living in neighbourhoods with the highest population density were found to be social factors associated with the mortality of older people diagnosed with COVID-19 in Europe;
- Immunosenescence and inflammaging are two important aspects of the aging immune system that contribute to immunological dysfunction in the elderly; another cause for ageing as a significant mortality risk is the increased prevalence of comorbidities in older adults.

### What this study adds

- We found that low oxygen saturation on admission was a risk factor for mortality in elderly COVID-19 patients;
- Corticosteroid therapy and anticoagulation were protective factors in elderly COVID-19 patients;
- To the best of our knowledge, this is the first study to examine the sociodemographic and clinical characteristics, mortality, and prognostic factors of COVID-19 patients over 60 years old in sub-Saharan Africa.
